# Social capital and fertility behaviors among female workers in healthcare settings: study protocol of a sequential explanatory mixed methods study

**DOI:** 10.1186/s12978-018-0507-6

**Published:** 2018-04-19

**Authors:** Mojgan Firouzbakht, Aram Tirgar, Karimollah Hajian-Tilaki, Abbas Ebadi, Fatemeh Bakouei, Maryam Nikpour, Mohammad Esmaeil Riahi

**Affiliations:** 1 Social Determinant of Health Research Center, Health Research Institute, Babol University of medical science, Babol, Iran; 2 Department of Biostatistics and Epidemiology, Babol University of Medical Science, Babol, Iran; 3 Behavioral Sciences Research Center. Life style institute, faculty of nursing. Baqiyatallah university of Medical sciences, Baqiyatallah University of Medical Sciences., Tehran, IR Iran; 4 Infertility and Health Reproductive Research Center, Health Research Institute, & Department of Midwifery, School of Medicine, Babol University of Medical Sciences, Babol, IR Iran; 5 Sociology of Health and Illness, Department of Social Sciences, University of Mazandaran, Babolsar, Iran

**Keywords:** Social capital, Fertility behaviors, Study protocol, Mixed methods design, Women

## Abstract

**Background:**

Fertility rate in Iran has decreased by more than 70% in the last three decades. Continuous decrease in fertility rate will create socioeconomic crises for the country in a near future. A significant factor behind fertility behaviors is women’s attitudes towards maternal and spousal roles. Such attitudes are mainly determined by social capital. This study aims to determine and explore of relationship between social capital and fertility behavior among female healthcare workers.

**Methods:**

This sequential explanatory mixed methods study will be conducted using the follow-up explanations model in two phases. In the first phase, a population-based cross-sectional survey will be conducted on 500 female workers recruited through multistage cluster sampling from healthcare settings located in Babol, Iran, and the relationship of social capital with fertility behaviors will be assessed. In the second phase, a qualitative study will be done to explain the findings of the first phase. Finally, the findings of the first phase will be explained using the findings of the second phase.

**Discussion:**

Understanding the relationship of social capital with fertility behaviors is essential to effective planning for the management of population decline. The findings of the present study will provide population policy-makers with helpful information.

## Plain English summary

Sever fertility decline in Iranian societies have made concern about aging population in future. The results of some studies showed that beside factors as modernization of societies and improved educational and employment of women, change in attitude of women about spousal and maternal role affected the fertility rate. Entering to the market has increased interpersonal relationship and social interaction of women. Interpersonal relationship form social capital, which have important role in individual attitude. This study is conduct to evaluate relationship between social capital and fertility behaviors among female workers in health care setting.

This study will have two phases. The first phase will be quantitative study. A cross section study will be conducted in 500 female workers in health care setting of Babol County. The instrument of the study will be questionnaires. The data will be analysis with statistical spss software. The results will demonstrate relationship of social capital and number of pregnancy, number of children and interval between children of participants.

The second phase of the study will be qualitative study with the conventional content analysis approach. The aim of this phase will be explain finding of quantitative study which need explanation. The data will be collected through participants’ interview and will be continue till data saturation. The data of this phase will be created themes. The themes are abstract concept which extract from statements of participants. Finally, an interpretation will be made to explain the quantitative finding based on qualitative finding.

## Background

Reduced fertility, improved living conditions, and increased life expectancy in recent years will result in the rapid increase of elderly population and the rapid decrease of human workforce in the following decades. This phenomenon will turn into a serious social challenge in most countries [1].

Like many other countries, population growth in Iran follows a downward trend [2]. Total fertility rate in Iran decreased from seven children per woman in 1980 to the replacement fertility level (i.e. 2.1 children per women) in 2000 and sub-replacement fertility level (i.e. 1.3 children per woman) in 2013 [3]. Estimates show that with a more than 70%-decrease in the past three decades, Iran has had the highest fertility decline rate in the world [4–6]. If remained unmanaged, such significant decrease will create socioeconomic crises for the country. In other words, the country will face the considerable needs of an aging population and will experience the same crisis currently experienced with respect to the young population [7, 8].

Decreased fertility rate in Iran is brought about by different socioeconomic factors. These factors include, but are not limited to, women’s improved educational and employment status, governmental policies for population control [2], religious authorities’ support for population control and increased marriage age [9, 10]. In the past, women had limited opportunities for education and employment and therefore, multiple births were a common practice among them. However, late in the twentieth century, women’s literacy level, financial independence, and political power considerably increased and they became more willing to enter job markets. Accordingly, significant changes happened in traditional employment patterns and also in women’s traditional housekeeping and childbearing roles in communities. These changes improved women’s educational and employment status and gave them greater power over their fertility behaviors. Women’s improved employment status on the one hand and their increased income on the other hand increased the opportunity cost of childbearing [11]. Therefore, women decided to postpone their fertility decision for the sake of their education or employment or both and hence, fertility pattern moved towards the one-child pattern [12, 13]. In other words, better educational and employment opportunities for women were associated with lower fertility rate [14]. All these changes show that one of the most significant factors behind reduced population growth is the changes in women’s fertility-related beliefs and attitudes.

Beliefs and attitudes are mainly determined by social interactions [15]. Women’s engagement in socioeconomic activities promotes their social interactions, which in turn affect their fertility behaviors. Through social interactions, women develop their fertility-related knowledge, get aware of others’ fertility-related experiences, and change their preferences and attitudes based on others’ viewpoints and attitudes [16]. Studies also showed that although fertility is a personal decision [17], it is greatly affected by social norms [18, 19].

People’s social interactions in social networks form their social capital. Social capital refers to the resources which people access through interpersonal relationships and social interactions. Such resources are accessible as needed and include stuffs, information, awareness, money, skills, power, or active support [20]. In fact, social capital is the product of interpersonal relationships in social networks and includes confidence, mutual understanding, shared values, and behaviors which connect the members of a network to each other, foster their collaboration [21, 22], and facilitate the gaining of mutual advantages [23]. The two main dimensions of social capital include structural and cognitive dimensions. The structural dimension refers to social networks, while the cognitive dimension includes norms and beliefs [22]. These two dimensions have causal relationships with each other [24].

Fertility-related social capital is informal resources which support parents to bear and rear their children. A study on 2016 women showed that accessible resources had positive effects on their intention for having their second or third child as well as on spacing between births [20]. Direct and indirect supports provided by social networks can also reduce the costs associated with fertility. These costs are both direct and indirect and include financial costs as well as the costs related to physical fatigue, heavier household workload, and changes in parental relationships. These costs can significantly affect individuals’ fertility intention [20, 25].

Given the continuing significant decreases in fertility in Iran, studies are needed to determine factors behind fertility intention and preferences. Most studies in this area have been done using either quantitative or qualitative designs. For instance, a cross-sectional study in Tehran, Iran, showed that 16% of the variance of women’s fertility-related attitudes was explained by sociocultural capital [26]. However, cross-sectional studies simply show that there are some relationships among variables and fail to show the direction of the relationships. Moreover, quantitative studies are not detailed enough to provide conclusive evidence for healthcare decision-making, planning, and policy-making. On the other hand, qualitative studies are not generalizable to large populations and their results cannot be used easily for operational planning. Mixed methods studies bring together the strengths of both quantitative and qualitative studies and provide a clearer understanding of the intended subject matter [27]. Therefore, the present study was designed and will be performed using a mixed methods study to determine and explore social capital and fertility behaviors among female workers in healthcare settings.

### Objectives

The objectives of the quantitative phase of the study are as follows:

- Assessment of social capital among female workers in healthcare settings;
- Assessment of fertility behaviors among female workers in healthcare settings; and
- Assessment of the relationship between social capital and fertility behaviors among female workers in healthcare settings.

The objective of the qualitative phase of the study

- Exploring female workers’ perceptions and experiences of fertility intention.

## Methods

**Fig. 1:**
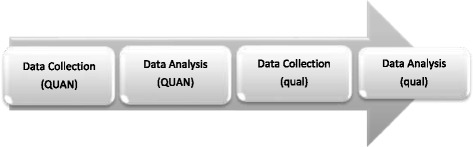
The schematic presentation of the study

This sequential explanatory mixed methods study with the follow-up explanations model will be conducted in two phases. In the first phase, i.e. the quantitative phase, data on female workers’ social capital and fertility behaviors will be collected using questionnaires and analyzed using statistical methods. Then, those quantitative findings which require explanation will be subjected to further exploration using a qualitative study in the second phase. It is worthy of note that in sequential explanatory mixed methods studies, quantitative and qualitative data are not independent from each other; rather, one is based on the other. In other words, the questions, sampling strategy, and data collection protocol in the qualitative phase are determined based on the findings of the quantitative phase. Finally, an interpretation will be provided to explain the findings of the quantitative phase using the findings of the qualitative phase. Therefore, the quantitative phase of this study will be more important to the aim of the study [28]. The phases of the present study are discussed in what follows and its schematic presentation is shown in Fig. 1.

### Phase I: The quantitative study

The first phase of the study will be a population-based cross-sectional survey to be conducted on female workers in healthcare settings located in Babol, a large city in the north of Iran.

#### Sample size and sampling strategy

Sample size was determined to be 500 women. Parameters for sample size calculation were a confidence level of 95%, a power of 80%, a social capital effect size of 0.2, and an attrition rate of 20%. Inclusion criteria are employment in hospitals or healthcare centers affiliated to Babol University of Medical Sciences, Babol, Iran, a work experience of at least 1 year, being married with at least one child, no previous history of primary or secondary infertility, and no history of system conditions which affect fertility (such as advanced cardiac problems or malignancies). Partial answering to data collection tools and voluntary withdrawal from the study will be the exclusion criteria in this study.

Participants will be recruited through multistage cluster sampling. Initially, the number of all female workers in the healthcare settings will be determined and then, the number of workers to-be-recruited from each setting will be calculated proportionately to the total number of female workers in that setting. Finally, participants will randomly be recruited from each setting. Accordingly, among the seven hospitals affiliated to Babol University of Medical Sciences, four will randomly be selected as clusters. Moreover, two healthcare centers will randomly be selected from either of the northern, eastern, western, and southern regions of Babol county—eight centers in total. Finally, eligible participants will randomly and proportionately be selected from the selected hospitals and centers.

#### Data collection tools

Quantitative data will be collected using the following four tools.

1. *A demographic questionnaire:* This questionnaire contains items on female workers’ age, marriage age, marital status, educational status, satisfaction with economic status, work experience, and shift work as well as their husbands’ educational and employment status.
2. *Women’s Fertility Behaviors Questionnaire:* Fertility behaviors in this study will be the actual number of children, the desired number of children, and birth interval. These behaviors together with their contributing factors will be assessed through the Women’s Fertility Behaviors Questionnaire which was developed by Taghizadeh et al. [29] based on the Fertility Changes Questionnaire [30].
3. *Bullen and Onyx’s Social Capital Questionnaire:* This questionnaire includes dimensions such as participation in social activities, relationships with friends, and relationships with family members. It will be used to assess social and support networks which are not related to participants’ work. The validity and the reliability of the Persian adaptation of this questionnaire were confirmed in an earlier study [31].
4. *The Workplace Social Capital Questionnaire:* As study participants are employees, this questionnaire will also be used to assess their workplace social capital. This questionnaire contains eight items and assesses social capital at personal and group levels in the two dimensions of participation and trust [32]. This questionnaire has not yet been used in Iran and therefore, we will assess its psychometric properties before its use. Accordingly, we will initially translate the questionnaire into Persian via the forward-backward translation method developed for the International Quality of Life Assessment Project [33]. Then, the face, content, and construct (convergent and discriminant) validity of the questionnaire will be assessed. The factor structure of the questionnaire will be assessed through confirmatory factor analysis. After that, reliability assessment will be performed through the internal consistency method with Cronbach’s alpha, McDonald’s omega, and theta value calculations as well as the test-retest stability method. In the test-retest stability method, 50 female workers from the study setting will complete the questionnaire two times with a 2-week interval and then, intraclass correlation coefficient will be calculated.

#### Data analysis

The SPSS (v. 21.0) and the Amos software will be employed for data analysis. Descriptive statistics measures will be used for the description of participants’ characteristics. Moreover, data analysis will be done through the Chi-square test, the one-way analysis of variance, Poisson regression analysis (for the number of children), survival analysis via Kaplan Maier and Long Rank analysis and Cox regression analysis (for the time of the first pregnancy), and single and multiple regression analyses. The significant level will be set as 0.05.

### Phase II: The qualitative study

The aim of this phase is to explain the findings of the first phase through a qualitative study with the conventional content analysis approach. In this approach, the data are explained and interpreted through immersing in the data and generating new concepts and categories from them. This approach is used when there is no theory or adequate empirical evidence in the intended subject area [34].

#### Sample size and sampling strategy

Participants will be recruited purposively based on the findings of the quantitative phase. In other words, we will determine those quantitative findings which need explanation, frame study question, and collect necessary data through interviewing some of the participants of the quantitative phase. Sampling will be done with maximum variation (respecting participants’ age, educational status, number of children, economic status, and organizational position) and will be continued until data saturation.

#### Data analysis

The data will be analyzed through the conventional qualitative content analysis. First, the recorded voice of each interview will be transcribed word-by-word and the transcript will carefully be perused to identify its manifest and latent contents. Then, words and expressions which are in some ways related to the study question will be coded and the codes will be grouped into subcategories and categories based on the similarities and the differences among them. Similarly, subcategories and categories will also be grouped into theme(s). Memos will be written in order to link subcategories, categories, and theme(s) to each other.

### Integration of quantitative and qualitative findings

After the completion of the quantitative and the qualitative phases, an interpretation will be made to explain the findings of the quantitative phase based on the findings of the qualitative phase. The phases of the study are summarized in Table 1.

**Table 1:** Diagram for this MMs study with the explanatory sequential design

Phase	Procedure	Product
Quantitative data collection	Cross-sectional study(N ≈ 500)	Numeric data
Quantitative data analysis	Data screening (frequencies, percent, poison regression, cox regression) using IBM SPSS software V.21	Descriptive statistics, linearity, multivariate outliers, hypothesis testing
Development of the Interview Protocol & Case selection	Purposeful recruitment based on typical response and maximal variation principle Developing interview questions	Case?
Collecting or making the qualitative data	Individual in-depth interview with participants (Until data saturation)	Text (non-numeric) data (interview transcripts)
Qualitative data analysis	Coding and thematic analysis Development of the within- case and across-case themes using the constant comparative methods (e.g. conventional qualitative content analysis)	Text data (interview transcripts) Codes, categories and themes
Integration of the Qualitative and the Quantitative Results	Interpretation and explanation of the quantitative and qualitative results simultaneously	Discussion Implications for the policy and the public future research

## Discussion

Fertility is not a simple biomedical experience; rather, it is a complex phenomenon with different biomedical, social, and cultural aspects and is affected by different factors. These factors may be specific to the immediate context [35]. Therefore, studies are needed to determine these factors. There are some studies about fertility decline in Iranian with quantities or qualities studied [1–3, 15]. Of course, cross-sectional studies with self-report data collection methods have some biases and provide no credible information about the direction of relationships among variables. Qualitative studies have very limited generalizability [36]. Therefore, their findings cannot be used for effective policy-making [37]. Mixed methods studies with both quantitative and qualitative methods can be used to minimize these weaknesses and provide more credible information. The present sequential explanatory mixed methods study will analyze the different aspects of the relationship between social capital and fertility behaviors. Its findings can be used for more effective healthcare planning and policy-making.

Among the strengths of this study is its sequential explanatory mixed methods design. This study will be done for the first time in Iran concurrently with new population policies. One limitation of this study will be participants’ reluctance to provide clear answers to items on private issues.
